# Data-driven RRAM device models using Kriging interpolation

**DOI:** 10.1038/s41598-022-09556-4

**Published:** 2022-04-08

**Authors:** Imtiaz Hossen, Mark A. Anders, Lin Wang, Gina C. Adam

**Affiliations:** 1 Department of Electrical and Computer Engineering, The George Washington University, Washington, DC 20052 USA; 2 National Institute of Standards and Technology, Gaithersburg, MD 20899 USA; 3 Department of Statistics, The George Washington University, Washington, DC 20052 USA

**Keywords:** Electrical and electronic engineering, Electronic devices

## Abstract

A two-tier Kriging interpolation approach is proposed to model jump tables for resistive switches. Originally developed for mining and geostatistics, its locality of the calculation makes this approach particularly powerful for modeling electronic devices with complex behavior landscape and switching noise, like RRAM. In this paper, a first Kriging model is used to model and predict the mean in the signal, followed up by a second Kriging step used to model the standard deviation of the switching noise. We use 36 synthetic datasets covering a broad range of different mean and standard deviation Gaussian distributions to test the validity of our approach. We also show the applicability to experimental data obtained from TiO_x_ devices and compare the predicted vs. the experimental test distributions using Kolmogorov–Smirnov and maximum mean discrepancy tests. Our results show that the proposed Kriging approach can predict both the mean and standard deviation in the switching more accurately than typical binning model. Kriging-based jump tables can be used to realistically model the behavior of RRAM and other non-volatile analog device populations and the impact of the weight dispersion in neural network simulations.

## Introduction

Many research advances in neuromorphic systems with emerging devices rely on the availability of accurate device models to quantify their algorithmic performance due to the limited availability and high cost of RRAM/CMOS tape outs. However, RRAM modeling is challenging given its complex multi-physics behavior^1^. Significant progress has been made to determine models relevant for different applications. For example, SPICE models use an underlying physical model and fitting parameters to experimental data to simulate the current vs. voltage characteristics useful for circuit design^2–8^. Some models focus entirely on physical principles^9–13^, but might be incomplete if they model only some specific behaviors or do not include the entire set of principles required to capture the multi-physics operation of the device. In general, physical model development for new devices requires assumptions regarding the underlying physical phenomena and the shape of the filaments^14–17^, which takes time to uncover and thus can delay the investigation in algorithmic simulations. Atomistic models based on first-order principles can provide significant insight into the device behavior and variability^18–26^, but are very computationally intensive and thus typically restricted to device investigations, not large scale neural network simulations.

By comparison, jump table models (or their variant increment plots) are derived only from experimental data and are agnostic to the underlying switching mechanism. They are phenomenological, stochastic lookup tables that define the probability of moving from one weight state to another. Since they are purely derived from data, they can represent a broad range of device non-idealities in network simulations^27,28^. While this jump table methodology can only predict the next conductance state and is not applicable for circuit modeling, it is particularly pertinent for modeling the weight update in neural network simulations. Switching noise leads to weight dispersion, which can impact negatively the accuracy and performance of the overall system^29,30^. Modeling this switching noise is therefore significant for providing a realistic estimate of the training of neuromorphic systems implemented with real devices.

Jump tables have been traditionally derived from experimental data using binning. However, this approach might not be statistically optimal and can introduce unwanted artifacts or exclude key device behaviors. We propose the use of a two-tier Kriging interpolation approach to model RRAM in the jump table framework and perform a statistical investigation into the validity of the proposed approach. Kriging interpolation, also known as Gaussian Process Regression process (GPR) has been previously used to separate signal and noise for analog memory elements for neuromorphic computing^31^. GPR was used to predict the noise-free signal (mean) in order to separate total variability into two parts: device-to-device variability and inherent switching variability for a device. The uncertainties in the readout and in the programming can also be modeled using linear fitting for readout and a sliding window and statistical correction for the programming data respectively^32^.

By comparison, our proposed two-tier approach models both the mean and the standard deviation for the device switching using Kriging interpolation to fully model the jump table. Our contributions are summarized below:

- The Kriging interpolation is functionally more suitable for continuous data, e.g., RRAM measurements, i.e., it does not introduce artifacts and artificial constraints on the data by comparison with binning and nearest neighbor approaches.
- A two-tier methodology was introduced, which models both the mean in the signal (first Kriging model) and the standard deviation of the switching noise (second Kriging model).
- The validity of the approach is tested by using 36 synthetic Gaussian data models. Our proposed Kriging interpolation approach can predict the synthetic distributions of the mean and standard deviation of device behavior with lower root mean square error (RMSE) than the binning and interpolation approach.
- The Kriging vs. binning approaches were also compared on experimental RRAM TiO_x_ device data using Kolmogorov–Smirnov and Maximum Mean Discrepancy tests. The experimental data was split into a modeling set and a test set to validate the approach. These tests compare whether two data sets are drawn from the same distribution and are critical for ultimately determining the utility of these approaches.

The remainder of the paper is organized as follows. “Device modeling” section introduces background information. “Methods” section III describes our method and “Results” section its evaluation on synthetic and experimental data sets. “Discussion” section concludes the discussion.

## Device modeling

### Jump table basics

Jump tables are cumulative distribution functions (CDF) of the change in device conductance per voltage (programming) pulse (ΔG/pulse) vs. initial device conductance (G). They can model the stochastic nature of the device programming and the nonideal conductance response ΔG as a function of initial conductance G^27–30^. The variability is represented by the standard deviation (SD) around the mean. An alternative representation in the resistance space (ΔR vs. R) called increment plots, is also possible^32^. However, for the remainder of this paper, the jump table in ΔG vs. G is used since the weight update is naturally mapped to the conductance space in a neural network implemented with real RRAM devices. When training a neural network, voltage pulses are applied to a device to adjust the conductance according to an equivalent desired weight during backpropagation^29^. Two jump tables are needed in simulation—one for potentiation (increase in G) and one for depression (decrease). For a device of initial conductance G, a probability value from 0 to 100% is generated from a uniform distribution using a random number generator. This probability value is used to determine ΔG/pulse from the CDF at conductance G. The device conductance is updated to G + ΔG and the cycle is repeated for follow-up pulses.

### Binning and interpolation

In typical jump tables, data binning is used to group individual data into bins of equal width or equal frequency^27^. From this binned data, the mean and standard deviation information can be extracted through a simple Gaussian fit. The mean and standard deviation values are then linearly interpolated across the G bins to obtain the jump table model.

**Figure 1:**
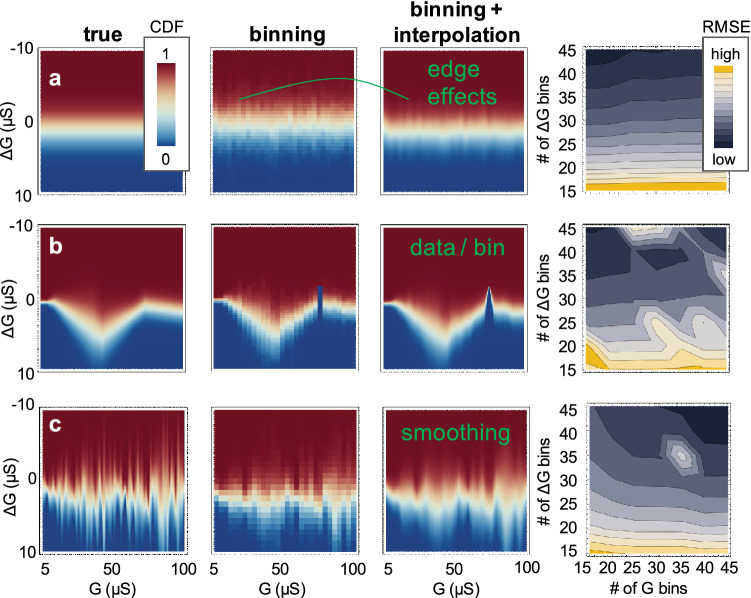
Binning challenges shown on synthetic distributions (**a**) with constant mean and SD; (**b**) an approximation of the model from ref^27^; (**c**) randomly generated mean and SD.

However, the appropriate number of bins for a given dataset may be difficult to optimize^33–35^ and prone to artifacts as Fig. 1 shows equal width binning for 3 representative synthetic datasets. The constant model requires a low G bin count for lowest RMSE, while the random one requires a high count. The underlying distribution of experimental data is unknown. Edge effects, empty bins and excessive smoothing due to the linear interpolation are other visible issues. These challenges motivate the search for a statistically sound alternative for jump table modeling, as well as methods to compare the predicted distribution against the measured dataset.

### Kriging/GPR modeling

The theoretical background for data modeling and prediction using Gaussian processes has been under development since the 1940’s^36^. In geostatistics, it is known as *Kriging* and applied particularly for two- and three- dimensional input variables^37–39^. However, it is also known more generally as Gaussian Process Regression (GPR) and applied for prediction purposes to a broad range of applications, from computer experiments^40^ to optimized machine learning algorithms^41^.

In Kriging/GPR, any collection of the responses of the data are assumed to be jointly Gaussian distributed with a mean function μ(x) defining the mean of the response *y* at any point *x* in the input space and a covariance function defining the covariance between the responses at any two points. Kriging predicts a function via a weighted average of the observed points using a set of considered functions as defined by the kernel of choice^42^. This technique has been used in RF device and analog circuit modeling^43–45^ as well as to extract mean behavior in phase change memory devices^31^.

While several types of Kriging modeling techniques exist, we utilize the ordinary Kriging in this paper, where the trend is constant. For interpolation situations, prior work^40,46,47^ shows that the constant trend plus a stationary Gaussian process in ordinary kriging can model complex systems as well as universal kriging where the trend is dependent on the variable. This paper assumes modeling only for interpolation purposes since typically the minimum and maximum conductance range for RRAM is fixed in neural network simulation. Since ordinary Kriging is computationally simpler, it is recommended to use in interpolation situations, such as our case.

Consider a RRAM measurement of $n$ sampled points $\left({G}_{1},\Delta {G}_{1}\right), \dots , ({G}_{n},\Delta {G}_{n})$, where for $i=1,\dots ,n.$ ${G}_{i}$ is the initial device conductance and ${\Delta G}_{i}$ is the corresponding conductance change. The ordinary kriging model with a noise term is described as

1 $$\Delta {G}_{i}=\mu +Z\left({G}_{i}\right)+{\epsilon }_{i},$$

where $\mu$ is the constant trend, ${\epsilon }_{i}\sim N\left(0,{\tau }^{2}\right)$^48^, and $Z(\cdot )$ is a stationary Gaussian process with zero mean and covariance function governed by a kernel $k$:

2 $$k\left({G}_{i}, {G}_{j}\right)=Cov \left(Z\left({G}_{i}\right),Z({G}_{j})\right){= \sigma }^{2}r\left({G}_{i}, {G}_{j}\right).$$

Here ${\sigma }^{2}$ is the process variance and $r\left({G}_{i}, {G}_{j}\right)$ is the correlation between the conductance change corresponding to two sampling points ${G}_{i}$ and ${G}_{j}$ for $i, j=1,\dots ,n$. The correlation function is typically chosen from a family of kernels functions, based on the application at hand. We chose an exponential kernel^42^ since it provided us with the lowest RMSE of the predicted mean. By the model in (1), we can obtain the parameter estimation $\widehat{\mu }={\left({1}^{T}{C}^{-1}1\right)}^{-1}{1}^{T}{C}^{-1}{\varvec{\Delta}}{\varvec{G}},$ where $1$ is a column vector of unity, $C=K+{\tau }^{2}{I}_{n}$, $K$ is the covariance matrix with entries $k\left({G}_{i}, {G}_{j}\right)$, ${I}_{n}$ is an identity matrix of order $n,$ and ${\varvec{\Delta}}{\varvec{G}}$ is the vector of $n$ sampled values for conductance change. Then the prediction of conductance change at a new value of conductance ${G}_{0}$ is given by

3 $$\Delta {\widehat{G}}_{0}=\widehat{\mu }+{R}^{T}{C}^{-1} (\boldsymbol{\Delta }{\varvec{G}}-\widehat{\mu })$$

where $R=(k\left({G}_{0},{G}_{1}\right),\dots ,k({G}_{0},{G}_{n}))$ is the row vector measuring the covariance between conductance change at ${G}_{0}$ and at the sampled points.

## Methods

### Datasets

**Figure 2:**
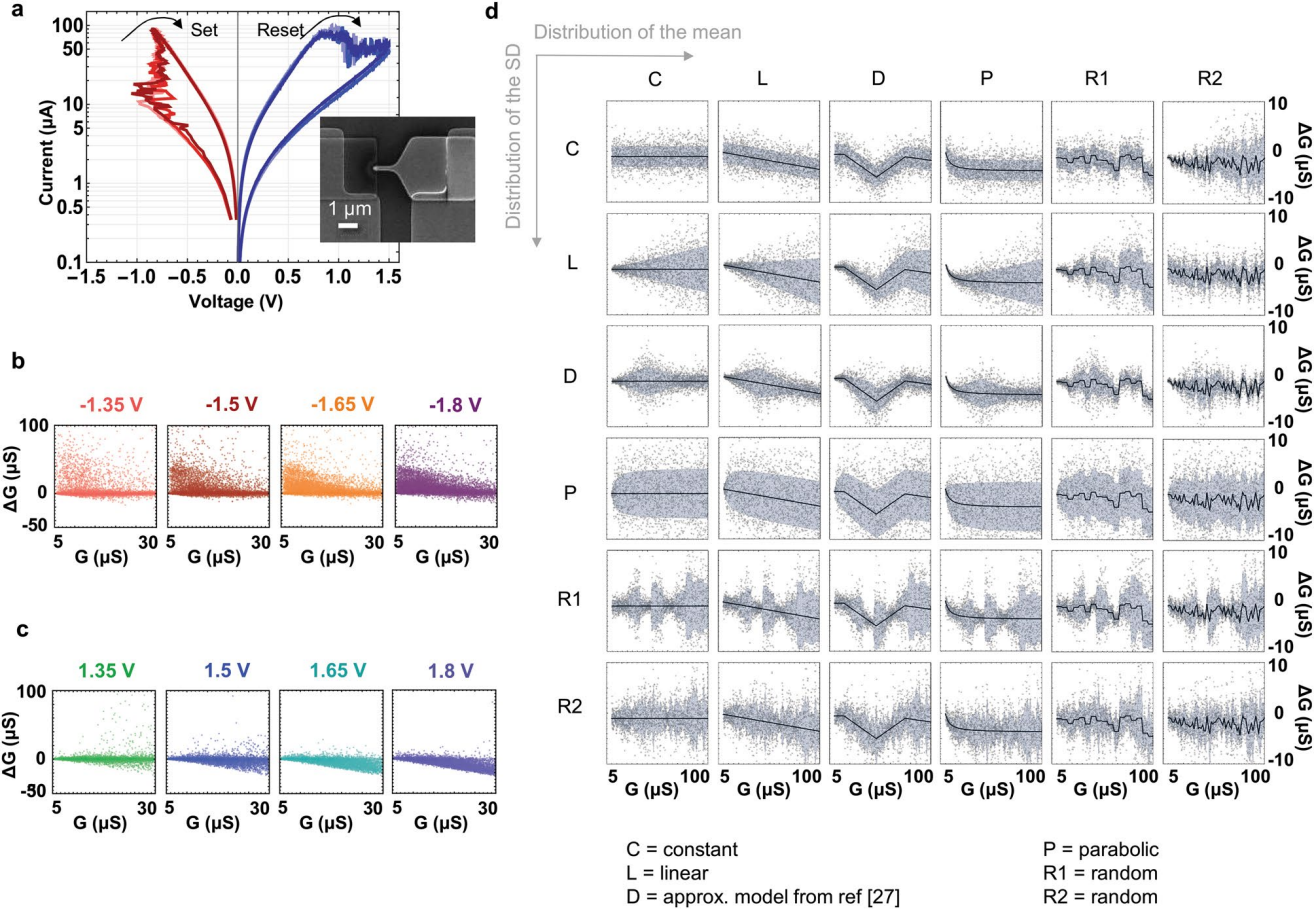
Device data. (**a**) RRAM used for experimental data gathering with current–voltage characteristics and SEM image; and ΔG vs. G data at different pulse amplitudes for (**b**) set and (**c**) reset. (**d**) Synthetic device data encompassing a wide range of distributions of mean and SD from constant to random.

This work demonstrates the proposed two-tier Kriging modeling vs. Binning approaches on two types of data (Fig. 2). Experimental data from a 10 nm edge device with 2.5 nm Al_2_O_3_/15 nm TiO_x_/5 nm Ti/30 nm Pt, was obtained utilizing two fast SMUs, a probe station, and text-based programming. The forming was done with monotonically increasing voltage pulses until the device was put into the high conductance state. Current–voltage measurements were made with a semiconductor parameter analyzer and are shown in Fig. 2a. The jump table data showed in Fig. 2b, c used for the modeling were collected after forming and then cycling the device 30 times for set and reset. The data collection algorithm is as follows. Before the algorithm starts, the device conductance is measured once. (1) The device is programmed to a random conductance value within a given range. (2) A write pulse is applied. The write pulse voltage is chosen randomly from a list. In this case, the list consists of ± 1.35 V, ± 1.5 V, ± 1.65 V and ± 1.8 V. All write pulses had a base voltage of 0 V, 500 ns high time, and 100 ns rise and fall time. (3) A subsequent read pulse of 100 mV is applied about 100 μs after the write pulse (during this time, the device is held at 0 V). The read pulse is held for about 20 ms and the current is measured and averaged for that time. G and ΔG values are recorded on each write pulse. 4) Steps 2 and 3 are repeated until either all pulses in the list have been applied or the device conductance exceeds some defined limit. 5) Return to step 1 and repeat the cycle. This algorithm was developed as it has some benefits over some other algorithms. For example, cyclically setting and resetting a device with monotonically increasing or decreasing write pulse voltage steps typically results in a sparse data set for high write voltages as the device typically will set or set before the higher voltages are applied. The algorithm described in this paper results in data sets of roughly equal number of points (~ 10,000) for each write pulse voltage. This work is based on experimental data obtained at a fixed reading voltage of 100 mV. The reason for that is because, in neuromorphic circuits, the read voltage is typically fixed for synaptic device programming^32^. However, RRAM devices present non-linear characteristics with the conductance change profile dependent on other factors beyond the initial conductance G. In the future, additional experimental data can be gathered, e.g. ΔG vs. G vs. write pulse width vs. write pulse amplitude vs. read-out voltage, etc. in order to devise a multi-dimensional model that can support other programming schemes.

However, it is important to point out that the proposed modeling approach and testing methodology can be applied to a broad range of RRAM devices based on other materials and with different (G, ∆G) switching properties. Jump table/increment plots modeling has already been applied to some phase change memory devices^27,29^ and to some TaO_x_ RRAM devices^28,30^ and to TiO_x_.

RRAM devices^32^. Kriging GPR was applied for signal vs. noise extraction in HfO_x_ RRAM devices and Ge_2_Sb_2_Te – based phase change devices^31^ as well as RF devices^43^ and nano CMOS thermal sensors^44^. Our proposed methodology can not only be applied for a more realistic modeling of RRAM jump tables, but it can also be applied beyond the ΔG vs. G switching of RRAM devices for other emerging devices where variability significantly affects behavior, e.g. spin torque transfer RAM^49^, Ferroelectric RAM^50^, conductive-bridge RAM^51^, two-dimensional materials based devices^52^, electrolyte transistor based synapses^53^, etc. and to other novel devices yet to be developed.

In order to be inclusive of the potential switching characteristics of such devices, we propose the use of synthetic data distributions with known means and standard deviations covering the full spectrum of statistics from constant to random distributions. In this work, 36 synthetic distributions with known means and standard deviations shown in Fig. 2d are used, exemplifying combinations of Gaussian distributions with 6 variable means and 6 variable standard deviation profiles for reset (depression) switching. For set (potentiation), positive mean values would be used instead. These profiles are as follows: constant (labeled as C); linear (L); piecewise linear (labeled D and inspired by IBM’s model from^27^); parabolic (P) and two random models (R1 and R2). For example, the constant model assumes a value of the mean ΔG of − 1 μS and of the standard deviation of 2 μS. At the other extreme, the random R2 model has randomly generated points between − 4.5 and − 0.5 μS for the mean profile and between 0.5 and 7 μS for the standard deviation profile. The synthetic (G, ∆G) data points are drawn from these Gaussian distributions, using a random number generator for a desired number of points. The detailed algorithm is listed below.

Our work explores a broad spectrum of synthetic device data, from constant to random profiles which goes well beyond actual devices that have been explored in existing literature. However, it is important to note that some of these profile shapes we discussed here seem to have been already observed experimentally. For example, the Ag:a-Si conductive bridge RAM modeled in the NeuroSim neural network simulator^54,55^ has a linear mean profile for both the set and the reset switching operations. The phase change memory device from IBM seems to have a piecewise linear behavior for the mean and standard deviation (see Fig. 17 in^27^). The TaOx RRAM devices modeled in^30^ seems to have a parabolic mean profile and a small constant standard deviation profile for the set, and somewhat random profiles for reset (see Fig. 12 in^30^).

While the focus of our work is on RRAM device modeling, the broader idea behind our synthesized data is to study how robust Kriging interpolation would be as a general modeling technique for various types of unconventional devices. Since the true profile of the mean and standard deviation is unknown for experimental data, these synthetic models were chosen to test the performance of the modeling approach across the entire range from constant to random. A constant profile should be easiest to predict, while a random profile the hardest. The chosen modeling approach should perform the best across the entire spectrum of profiles.
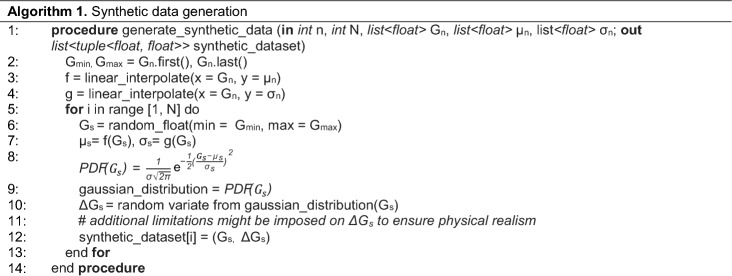


### Modeling and testing approach

**Figure 3:**
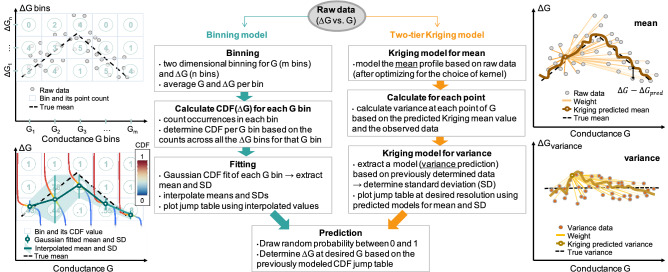
Algorithmic comparison. A binning modeling approach vs. the proposed two-tier Kriging modeling approach.

Figure 3 shows the algorithmic comparison between the typical binning modeling and the proposed two-tier Kriging modeling approach. As a first step, a one-dimensional ordinary Kriging is used to predict the mean at each G based on the raw data. The variance of the sampled data to the Kriging mean is determined for each point and used to extract the variance ($\Delta G-{\Delta G}_{Krig}{)}^{2}$ at each point of G in the dataset. The second step consists of another one-dimensional ordinary Kriging, used to predict the standard deviation based on the previously calculated variance data. This modeling was performed with the DiceKriging package^56^ from R. By comparison, data binning is used to group individual data into regions of the designated size and extract mean and standard deviation information through a simple Gaussian fit. Based on Fig. 1, 30 bins for G and 40 bins for ΔG are used for binning in the remainder of the paper.

For the synthetic datasets with known mean and standard deviation, the root mean square error (RMSE) is calculated for the predicted mean and standard deviation, respectively for both approaches for various sample sizes. For the experimental data, the true underlying mean and standard deviation profiles are unknown. Therefore, it is not possible to calculate the mean of RMSE and SD from the experimental data. We used the Kolmogorov–Smirnov (K–S) test and the maximum mean discrepancy (MMD) test to determine the goodness of fit between the observed distribution and the predicted distribution. The K-S test is a non-parametric test that can compare a sample drawn from an unknown distribution to a known reference continuous distribution^57,58^ or it can estimate the maximum distance ${D}_{n}={\mathrm{sup}}_{\mathrm{x}}|{F}_{P}(x)-{F}_{Q}(x)|$ between two empirical cumulative distributions $F$ in our case of the predicted dataset based on the modeled experimental data $P$ vs. the experimental test dataset $Q$. Since the K-S test tends to be less sensitive at the tails of the distributions among other limitations^59^, we utilized MMD as a complementary measure. MMD^60^ represents the distance between empirical distributions as the distance between their mean embeddings ${\mathbb{E}}$ determined via feature maps $\varphi$ (or reproducing kernel Hilbert space $\mathcal{H}$) $MMD (P\left(x\right),Q\left(y\right))={\mathrm{sup}}_{{\Vert \varphi \Vert }_{\mathcal{H}}\le 1}|{\mathbb{E}}_{{\varvec{P}}}\left[\varphi \left(x\right)\right]-{\mathbb{E}}_{{\varvec{Q}}}[\varphi \left(y\right)]|$. The kernel determines the type of distance computed. Some, e.g. Gaussian kernel, lead to the MMD distance being zero only if the two datasets are drawn from the same underlying empirical distribution. MMD provides the maximum distance across the test kernels. Two R packages^61,62^ are used.

## Results

**Figure 4:**
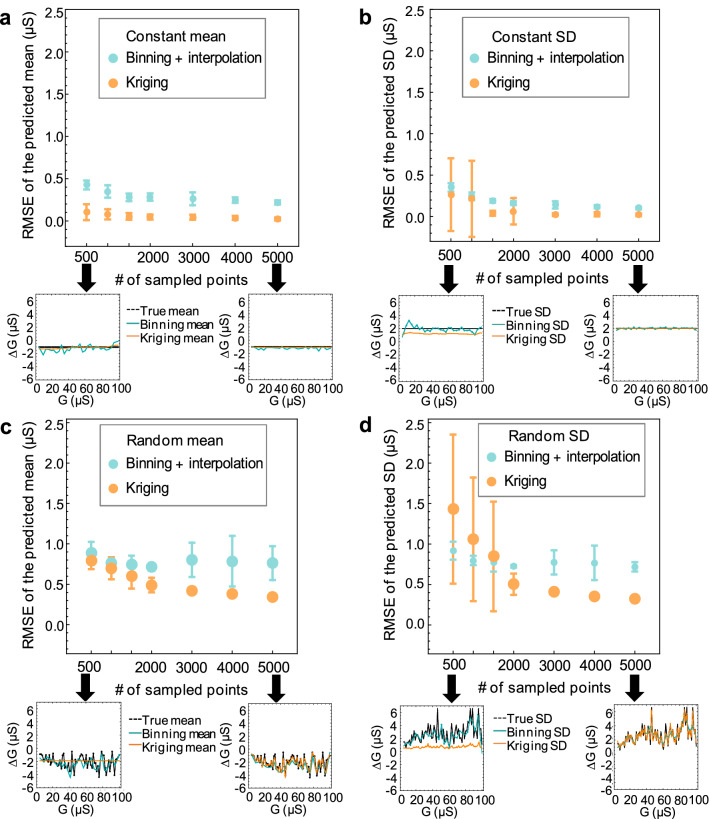
Prediction RMSE of Binning and Kriging model vs. number of sampled points in the dataset. (**a**, **b**) prediction of the mean and SD for the constant distribution model and (**c**, **d**) for the random distribution model R2. # of iterations = 20 and the error bar indicates one standard deviation.

Figure 4 shows the impact of the sample size on the RMSE of the mean and standard deviation prediction of the two approaches on two synthetic models—constant mean and constant standard deviation (C) and random mean/random standard deviation (R2). The insets are showing worst case scenario for the Kriging prediction of the mean and standard deviation profiles. Overall, Kriging predicts the mean and the standard deviation profiles better than the Binning approach, particularly at higher sample count. Kriging has difficulty robustly predicting the desired profile with a low sample count as shown by the large error bars for the estimates with less than 2000 points. This is most evident in the mean/standard deviation predictions of the random model for 500 points in the sampled dataset (Fig. 4c, d). For that case, the Kriging model is predicting them wrongly as (close to) constant although the true mean and standard deviation profile have random behavior (dashed black line). However, at larger sample sizes, the Kriging interpolation consistently estimates the mean and the standard deviation profile better than the binning approach. For example, for the random model at 5000 data points, the Kriging RMSE for the mean prediction is 0.345 ± 0.033 μS vs. for the binning 0.76 ± 0.021 μS and the RMSE for the standard deviation Kriging prediction is 0.326 ± 0.024 μS vs. the RMSE of 0.072 ± 0.06 μS for the Binning. This indicates the existence of a minimum number of points required to generate an accurate model for high randomness in the mean and SD. Large datasets are desirable, but difficult to obtain in practice and computationally intensive for the Kriging approach. Our results indicate that samples with at least 2000 points should be used.

**Figure 5:**
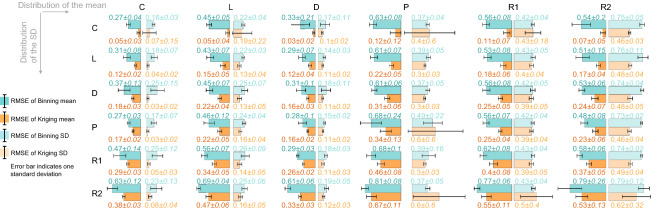
Comparison of mean and SD prediction results for the 36 synthetic models. The Kriging interpolation for the mean is consistently better than binning approach. # Points = 2000, # G bins = 30, # ΔG bins = 40, # of iterations = 20.

Figure 5 shows the results across all 36 synthetic data models. As the complexity of the mean/standard deviation profile increases, the RMSE increases as expected for both binning and Kriging. The lowest errors are seen for the constant, linear and the device model from^27^. The models with parabolic mean and/or standard deviation distributions also seem to perform poorly. This may be because both approaches are based on linear interpolation, weighted or not. Kriging interpolation consistently performs the best for the mean prediction, but there are a few instances when it underperforms for the parabolic and random standard deviation cases.

**Figure 6:**
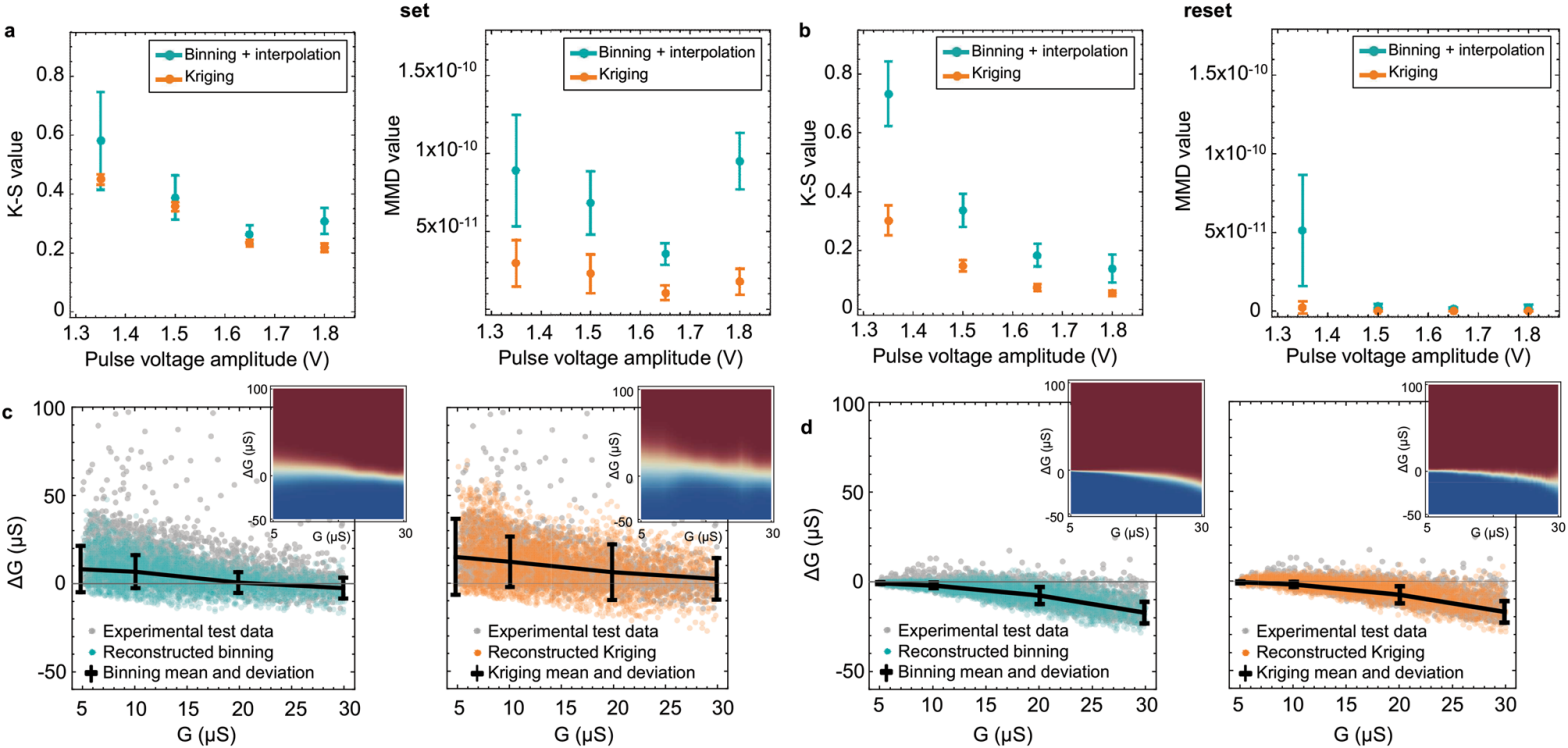
Results for experimental test data. K–S test and MMD metrics of test data vs. reconstructed data for four pulse amplitudes (**a**) set and (**b**) reset. # Points = 4000 experimental/reconstructed data points, #iterations = 20. Reconstructed data based on Binning + interpolation and Kriging modeling for (**c**) set (**d**) reset at pulse amplitudes ± 1.8 V.

Both methods were applied to the experimental data of TiO_x_ RRAM devices for various pulse amplitudes. A random sub-sample of 4000 data points (modeling set) was used to generate the Binning and Kriging models for set and reset respectively. Another non-overlapping 4000 points sub-sample (test set) was compared with the predicted models. The K–S and MMD tests were used to provide a quantitative estimate of the difference between the predicted and experimental test sets, as reference. The experimental data (as shown in Fig. 2) was randomly sub-sampled for the modeling and testing sets and modeled using this methodology 20 times to provide statistically significant and computationally accessible results. Both the set and reset results are shown in Fig. 6. The lower value of K–S and MMD indicate the better model fit to the experimental test data. On average, the K–S and MMD values of Kriging reconstructed data are less than those of binning + interpolation based reconstructed data. Both binning and Kriging models give a better prediction for reset data (Fig. 6b) than the set data (Fig. 6a). Kriging K–S values for reset.

are almost half in comparison with the K–S values for set. However, the standard deviation of K–S and MMD values for Kriging is lower than the binning + interpolation which can indicate that the reconstructed data of the Kriging model is more consistent than the reconstructed data of binning + interpolation method.

It is also important to notice that for the set data, there are 18 cases out of 80 for which the binning + interpolation method leads to slightly better results than the Kriging model, particularly at lower pulse voltage amplitudes. This might be because the standard deviation prediction is likely affected since our models assume a Gaussian distribution whereas the set data seems to be intrinsically skewed. To test this hypothesis, Fig. 6c, d includes reconstructed data vs. experimental data for set and reset respectively at pulse amplitudes ± 1.8 V. As shown in Fig. 6c, both binning + interpolation and Kriging models have some difficulty reproducing the overall shape of the set data due to skewness. However, the binning underestimates the standard deviation by a larger margin than the Kriging, as seen in the two jump tables (insets). Based on these quantitative and qualitative results, the reconstructed data from the Kriging model seems to cover better the test experimental data.

## Discussion

**Figure 7:**
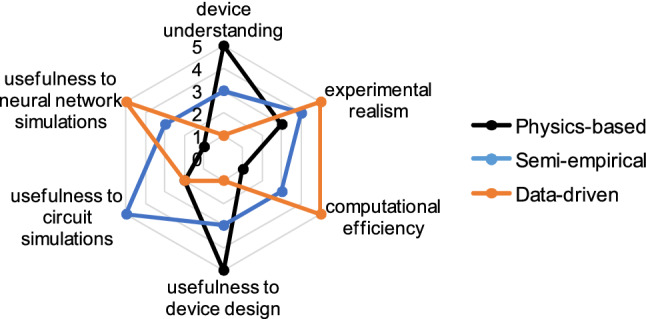
Comparison of key considerations for the major categories of device modeling approaches. The proposed two-tier Kriging approach can be classified as a data-driven device modeling approach.

The applicability of the proposed modeling approach has to be discussed in the broader context of RRAM device modeling. Three broad device model categories can be considered: physics-based models, semi-empirical device models and empirical (data-based) models, in accordance with existing categorizations in the field at large^63,64^. The proposed methodology belongs in the last category of data-driven models and its advantages and limitations are highlighted in Fig. 7. Physically-based models are supported by first-order principles and fundamental calculations. They can provide accurate representations via atomistic simulations or closed form solutions of coupled nonlinear partial differential equations. This can significantly improve the understanding of the internal physical phenomena and support robust device design^65^. However, these approaches are typically computationally intensive and might be incomplete and unable to fully explain the experimental reality. By comparison, semi-empirical (or physically-based compact) models are particularly useful for circuit design. These compact models are typically based on a simplified physical model with fitting parameters to make the model conform to experimental current vs. voltage data. Since only a few equations have to be solved at each step, the computational efficiency is improved and with careful fitting, these models are particularly useful in circuit simulators, e.g. SPICE^66^. However, for large neural network simulations that require constant device update modeling, these compact models can be insufficient. Large-scale models can require thousands to millions of individual conductance updates per training epoch depending on the batch size and network size, which requires accessing the device model the same number of times. In these situations, semi-empirical models can be too computationally intensive^45^. Data-based models, such as the one proposed, aim to provide a fast approach to derive realistic models of novel devices that might otherwise be difficult to represent by physical functions.

Since data-based models are generated directly from measured data without knowledge of the device physics, the fitting methodology used is entirely responsible for capturing the features of the data. In this work, we have showed that suitable statistical methods, e.g. Kriging/GPR should be used for continuous RRAM data since they tend to perform better than binning approaches. Moreover, the validity of the approach has to be tested using appropriate methods, e.g. on synthetic datasets with known distributions, using nonparametric tests for sample comparison, etc. It is important to point out that our two-tier modeling approach based on ordinary Kriging is suitable for interpolation, in the range between minimum and maximum conductance of the experimental data, as needed for neural network simulations using these device models. For extrapolation situations, the functional type assumed for the trend model is very important, so if extrapolation needs to be considered, more sophisticated Kriging (GPR) methods such as Universal Kriging have to be considered.

This work also highlights a potential limitation of the proposed approach for skewed data and the need to expand to more general non-Gaussian interpolation methods. Existing literature models variability as a Gaussian distribution, for example the jump tables by Burr et al. seem to also have this assumption^27,29^. The uncertainty model of switching noise by Stathopoulos et al. also assumes Gaussian data distributions for its RRAM devices^32^. Gong et al. established a practical method to separate the signal and noise components of analog NVM elements based on the Gaussian regression process^31^. Perez et al. also assumes a Gaussian distribution for the readout current vs. read voltage of RRAM devices^67^. However, our results show that the skewness can be a challenge, particularly for set operation without compliance. The skew can have many potential origins, an obvious example being the physical bounds on the maximum amount that the conductance can be changed, e.g. the conductance cannot go above the ceiling set by the series parasitic resistance. Or if the initial G is 35 μS, the reset ΔG cannot be < -35 μS, which we considered in how we reconstructed the Binning/Kriging sets. Non-Gaussian methods that support the incorporation of physical constraints would be desirable for the modeling of non-volatile memory devices.

Another limitation is the fact that current–voltage characteristics are not modeled and cannot be reconstructed as part of this work. The lack of physical insight into the actual device can also be considered a drawback. However, it is important to note that GPR has been previously used to predict the mean signal and separate device-to-device variability and cycle-to-cycle variability^31^. The series resistance could also be potentially estimated based on the lower data bound in reset data. These can be critical insights drawn entirely from measured data and useful for device optimization. However, if voltage-behavior is incorporated in the model, it can be useful for fitting important parameters for compact models, e.g. the evolution curve of read resistance vs. pulse amplitude typically fitted with ad-hoc functions and fitting parameters^1^. The resulting model of read resistance change could model the dynamic behavior of the device and together with a physically inspired static model could reproduce current–voltage RRAM characteristics. In addition, the proposed technique has potential to also be applied in contexts relevant to SPICE modeling where the focus is on current vs. voltage characteristics for circuit simulation of inference operations. For example, a behavioral model was recently proposed for multi-state HfO_2_-based memristors by modeling CDFs of the readout currents at five conductance states via simulation in LTspice^67^. In that work, experimental CDFs of the measured readout currents were fitted with a Gaussian distribution, while the means and standard deviations as a function of read voltage were linearly fitted. According to the authors, the specific implementation of this model is a SPICE sub-circuit with two terminal connections and a parameter for the selection of the device conductance state. The model was successfully used in circuit simulations of neural networks^68^. Kriging modeling could be potentially applied to model the means and standard deviations of the readout current as a function of the read voltage instead of the simple linear fit. Moreover, the Kriging/GPR can be expanded to multi-dimensional data that include pulse width, pulse amplitude, read out voltage, temperature, etc. thus providing a comprehensive model capturing the device population behavior. This has direct applicability to identifying the most optimal programming scheme for the devices which can reduce the experimental variability observed^68^, and thus more realistically modeling RRAM-implemented weight updates in neural network simulations.

Nevertheless despite the targeted applicability to memristive weight update modeling, it is important to point out that the proposed modeling approach and testing methodology can be extended to modeling a broad range of novel non-volatile memory devices in a prompt fashion conducive to large-scale neuromorphic simulations and experimental demonstrators. As soon as the (G, ∆G) switching properties are measured, the two-tier Kriging model can be obtained and incorporated in a simulator, e.g. NeuroSim^55^. The proposed methodology for testing can be utilized to determine the goodness of fit between the observed experimental distribution and the predicted distribution based on the model. A small distance between these two distributions is desired to ensure that the simulation results are indicative of potential performance results on a hardware prototype.

## Conclusions

This paper proposes a two-tier Kriging/GPR approach for modeling jump tables of RRAM devices and tests it comparatively to the traditional binning approach using a broad range of synthetic Gaussian datasets with known mean and standard deviation profiles, as well as experimental data. Binning introduces artifacts and artificial constraints to continuous data. The Kriging modeling can determine the data trends more reliably providing a better prediction than the binning for all the investigated mean models and almost all standard deviation models. The work also demonstrates the use of statistical tests e.g. K-S and MMD, to determine how far the reconstructed points based on the proposed models are from the underlying experimental data. This work also highlights that for skewed experimental data, the Kriging model can fail when the assumption of Gaussian distribution is no longer valid.

Future work will expand to non-Gaussian methods that consider physical constraints to better predict the experimental data and to generate reliable statistical models of analog device dynamics for use in neuromorphic simulations. The performance advantage is expected to be larger for higher dimensionalities of the parameter space which will also be explored in the future. Multivariate distribution-free two sample tests will be essential for determining the suitability of such methods.
